# A Systematic Review of the Evidence for Non-surgical Weight Management for Adults with Severe Obesity: What is Cost Effective and What are the Implications for the Design of Health Services?

**DOI:** 10.1007/s13679-022-00483-z

**Published:** 2022-11-21

**Authors:** Elisabet Jacobsen, Dwayne Boyers, Paul Manson, Alison Avenell

**Affiliations:** 1 Health Economics Research Unit, University of Aberdeen, Polwarth Building, Foresterhill, Aberdeen, AB25 2ZD UK; 2 Health Services Research Unit, University of Aberdeen, Aberdeen, UK

**Keywords:** Severe obesity, Weight management programmes, Systematic review, Cost-effectiveness

## Abstract

***Purpose of Review*:**

Severe obesity (BMI ≥ 35 kg/m^2^) increases premature mortality and reduces quality-of-life. Obesity-related disease (ORD) places substantial burden on health systems. This review summarises the cost-effectiveness evidence for non-surgical weight management programmes (WMPs) for adults with severe obesity.

***Recent Findings*:**

Whilst evidence shows bariatric surgery is often cost-effective, there is no clear consensus on the cost-effectiveness of non-surgical WMPs.

***Summary*:**

Thirty-two studies were included. Most were short-term evaluations that did not capture the long-term costs and consequences of ORD. Decision models often included only a subset of relevant ORDs, and made varying assumptions about the rate of weight regain over time. A lack of sensitivity analyses limited interpretation of results. Heterogeneity in the definition of WMPs and usual care prevents formal evidence synthesis. We were unable to establish the most cost-effective WMPs. Addressing these limitations may help future studies provide more robust cost-effectiveness evidence for decision makers.

**Supplementary Information:**

The online version contains supplementary material available at 10.1007/s13679-022-00483-z.

## Introduction

In England, 29% of adults have obesity (body mass index (BMI) ≥ 30 kg/m^2^) [1], whilst at least 7% of men and 9% of women have severe obesity (which we define as BMI ≥ 35 kg/m^2^) [2]. Obesity-related diseases (ORDs) such as type 2 diabetes mellitus (T2DM), cardiovascular diseases, stroke, and obesity-related cancers reduce life expectancy [3] and are detrimental to patient health and quality of life. The economic burden of obesity in England is projected to be approximately £16 billion per year [4]. In 2017/2018, 711,000 hospital admissions were associated with obesity, an increase of 15% from the previous year, demonstrating that obesity is a growing health concern [1].

Economic evaluations are comparative analyses of the costs and benefits of different health care interventions and provide information to help decision makers reach evidence-based decisions on the efficient allocation of scarce health care funding resources. International decision makers, such as the National Institute for Health and Care Excellence (NICE) in the UK and Canadian Agency for Drugs and Technologies in Health (CADTH) in Canada provide funding recommendations on the use of health technologies using economic evidence as an integral part of their decision-making processes. For example, in the UK, NICE published obesity guidance in 2014 [5] that recommended a weight management programme (WMP) for people with obesity, pharmacotherapy if WMPs had failed, a very low calorie diet (VLCD) for people that needed to lose weight quickly (such as for infertility treatment or joint replacement) and bariatric surgery for those with a BMI ≥ 40 kg/m^2^ and BMI of 35–40 kg/m^2^ for people with comorbidities.

Despite the substantial health, social and economic burden, there remains a lack of evidence synthesis that clarifies the most effective and cost-effective management strategies for people with severe obesity (and their comorbidities). The aim of this paper is twofold. First, we report the findings of existing cost-effectiveness studies evaluating non-surgical WMPs for people with severe obesity. Secondly, we identify common evaluation challenges, with a view to providing recommendations for the conduct of future obesity economic evaluations.

## Methods

### Search Strategy

We searched MEDLINE and EMBASE databases from 1980; NHS Economic Evaluation Database (NHS EED), Health Technology Assessment (HTA) database, Cost-effectiveness Analysis Registry, and Research Papers in Economics (RePEc) from inception. Original searches by us up to May 2017 were conducted as part of the REview of Behaviour And Lifestyle interventions for severe obesity: AN evidenCE synthesis (REBALANCE) study [6••]. Updated searches were conducted up until November 2020. Full details of search strategies are provided in our REBALANCE report [6••].

### Inclusion and Exclusion Criteria

English language studies, reporting full economic evaluations, defined as a comparative assessment of two or more non-surgical WMPs (i.e. cost-utility analysis (CUA), cost-effectiveness analysis (CEA), cost–benefit analysis (CBA) or cost-minimisation analysis (CMA) frameworks) were deemed eligible for inclusion. Eligible populations were adults aged 18 and over, with severe obesity (BMI ≥ 35 kg/m^2^) based on mean or median BMI in source clinical effectiveness studies (or a modelled cohort with (BMI ≥ 35 kg/m^2^)). Interventions were eligible for inclusion so long as they were a WMP, where the key target of the intervention was weight loss or weight loss maintenance. This also included VLCDs, defined here as ≤ 800 ± 10% kcal/day. Partial economic evaluations such as evaluations of costs alone or outcomes alone, cost-consequence analyses (costs and consequences not compared but reported separately) and methodological studies were all excluded. The only pharmacotherapy included was Orlistat because, at the time of writing, it was the only drug prescribed for weight loss in the UK.

### Data Extraction

Abstract screening was conducted by one health economist. Full texts were evaluated against the inclusion and exclusion criteria and checked by a second health economist for consensus. All included studies were data extracted into a predefined online data extraction form. The data extraction form for our REBALANCE review was designed to include all economic data available within the studies, but in the updated review, a targeted data extraction form was used, extracting only data required for the current article [7]. The updated data extraction form is provided in the Supplementary Material Table 1.

### Narrative Evidence Synthesis

Findings from the systematic review were tabulated, and a narrative synthesis of the cost-effectiveness evidence provided. Data were not synthesised quantitatively due to substantial heterogeneity across included studies in terms of evaluation frameworks (CUA, CEA), evaluation approach (within trial evaluations or decision models), scope of evaluation (narrowly defined such as diabetes vs broadly defined multiple ORDs), differences across health care systems, definitions of interventions and comparators. Methodological limitations of the studies were identified and catalogued, with a view to providing guidance for future research.

### Quality Assessment

Included studies (in our REBALANCE report [6••]) were quality assessed using standardised checklists, recommended by Cochrane: economic evaluations (EEs) alongside clinical trials and decision analysis models used Drummond and Jefferson [8] and Philips et al. [9] checklists, respectively. Quality assessment was done independently by two health economists for the individual review, the results of which can be found in the REBALANCE report [6••].

Studies identified in this updated review were assessed against the methodological issues identified in the REBALANCE review to identify whether the quality of studies has improved over time.

## Results

### Identified Studies

**Fig. 1:**
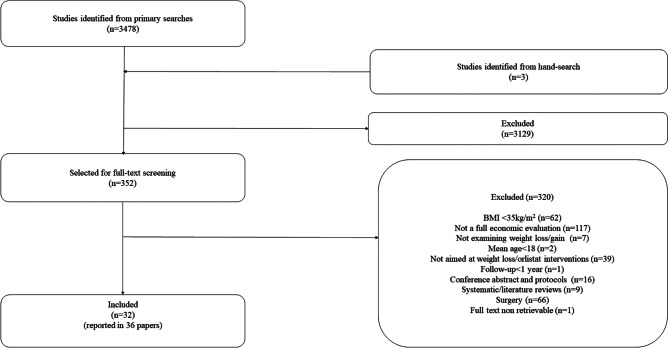
PRISMA flow chart for identification of studies from 1990 to 2020

The searches, combined for the original and updated reviews, identified 3478 potentially relevant titles and abstracts. *N* = 352 full texts were retrieved and assessed against the inclusion/exclusion criteria*. N* = 32 studies were finally included in the review (reported in 36 papers). Further details are provided in the PRISMA flow chart (Fig. 1).

Economic evaluations included evaluations of WMPs (*n* = 29) and pharmacotherapies (*n* = 5). Two studies evaluated both WMPs and pharmacotherapies [10, 11]. These are listed in Table 1 and categorised in three groups: economic evaluations alongside randomised controlled trials (RCTs) (*n* = 13), others (neither RCT-based nor model-based) (*n* = 4) and decision models (*n* = 15). The majority of studies were published within the past 10 years (*n* = 29), and the remainder were published in 2005 (*n* = 3). The WMPs are further categorised as lifestyle WMPs (*n* = 25) [6••, 10–22, 23••, 24•, 25, 26•, 27–32, 40], VLCDs (*n* = 4) [6••, 26•, 27, 29], meal replacements (*n* = 2) [10, 11], group intervention (vs intervention delivered on individual basis) (*n* = 1) [33], and remote interventions (*n* = 6) [12–14, 34–36]. Five studies included Orlistat in their assessment (*n* = 5) [10, 11, 37–39]. Some studies evaluated multiple interventions and therefore a study can have multiple WMP categories. The WMP categories are listed in Table 1, the study characteristics table.

**Table 1:** Study characteristics

**Study**	**Country** **Setting**	**Population mean BMI**	**Description of intervention**	**Description of control**	**Intervention type**	**Primary treatment effectiveness source (follow-up in years; model time horizon in years)**^**f**^	**Costing perspective**	**Cost year (currency)**	**Discount rate-costs; benefits**	**Primary economic outcome measurement**	**Model description (details)**	**Health states model **^**a**^
**WMP–economic evaluation alongside RCT**												
Daumit et al. [12]	USA Community and home setting	People with obesity and at least one cardiovascular disease 37 kg/m^2^	In person versus remote sessions with weight loss coaches that focused on DASH reduced calorie diet aiming for 5% weight loss and physical activity advice, website access	Session with weight loss coach at randomisation and at final follow-up at 24 months if desired, website access	Lifestyle WMP: In person v control Remote intervention v control In person v remote	RCT (2; N/A)	Health care payer	2016 (US$)	NR	Cost per kg lost	N/A	N/A
Delahanty et al. [13]	USA Community and home setting	People with T2DM and are overweight/obese 35 kg/m^2^	Two intervention groups: in-person group or telephone group (conference calls) given by dietitian based on Look AHEAD material. Meal replacements (1–2 meals per day from week 3) were recommended. Participants also offered 5 individual sessions	Usual care defined as referral to dietitian	Lifestyle WMP: In person v referral to dietitian Remote intervention v referral to dietitian In-person v referral to dietitian	RCT (1^b^; N/A)	Third party payer	2018 (US$)	N/A	Cost per kg lost Cost per person achieving 2%/5% weight loss	N/A	N/A
Hollenbeak et al. [33]	USA Primary care practices	People with obesity and metabolic syndrome, without diabetes 39 kg/ m^2^	WMP based on Diabetes Prevention Programme with group conference phone calls	WMP based on Diabetes Prevention Programme with individual phone calls	Group v individual	RCT (1; N/A)	Society	2013 (US$)	N/A	Cost per QALY	N/A	N/A
Little et al. [14]	UK Primary care practices	People with BMI ≥ 30 kg/m^2^ (or ≥ 28 kg/m^2^ with risk factors) 37 kg/ m^2^	E-learning (with and without face-to face support). Physical activity advice with low carbohydrate (< 50 g/day) or deficit of 600 kcal/day	Brief verbal and online healthy eating advice	Lifestyle WMP: Remote v control In-person v control	RCT (1; N/A)	NHS and PSS	2013–2014 (GBP)	N/A	Cost per QALY	N/A	N/A
McKnight et al. [15]	USA Hospital based	Diabetes prevention programme: risk of type 2 diabetes, as defined by HbA1c ≥ 5.7% or a BMI ≥ 25 kg/m^2^ 36 kg/m^2^	Original Fit for LIfe (FFL) diabetes prevention programme on nutrition and exercise: 12 weekly sessions of 90 min	Fit for Life (FFL) diabetes prevention programme: 12 weekly sessions of 90 min, and an additional 3 individual counselling sessions and 3 personal training sessions	Lifestyle WMP: Lifestyle WMP	Before and after non-randomised study (12 weeks; N/A)	Intervention (programme) cost	2016–2017 (US$)	N/A	Cost per kg lost	N/A	N/A
McRobbie et al. [16]	UK Primary care practices	People with BMI ≥ 30 kg/ m^2^ or BMI ≥ 28 kg/ m^2^ with comorbidities 35 kg/m^2 c^	Weight Action Programme with healthy eating and physical activity advice	Four practice nurse sessions over 8 weeks, follow-up at 6 and 12 months	Lifestyle WMP	RCT (1; N/A)	NHS and social services	2012–2013	N/A	Cost per QALY	N/A	N/A
Meenan et al. [17]	USA Community centres	BMI ≥ 27 kg/m^2^ and taking antipsychotic agents 38 kg/m^2^	DAS-based diet (≤ 30% fat and ≤ 10% sat fat calories, for 4.5–6.8 kg weight loss) and exercise programme	Usual care (further described in study as no intervention)	Lifestyle WMP	RCT (1; N/A)	Health system or payer	NR	N/A	Cost per kg lost	N/A	N/A
Patel et al. [18]	UK Primary care	People with BMI ≥ 30 kg/m^2^ 36 kg/m^2 c^	Primary care-led behavioural intervention Ten Top Tips (10TT) self-guided leaflet with 10 weight loss tips, focusing on diet and physical activity, one primary care session, self-monitoring taught	Usual care: primary care usual practice, e.g. might include referral to dietitian, or Weight Watchers	Lifestyle WMP: Very low-dose WMP v usual care	RCT (2; N/A)	NHS and personal social services (PSS)	2013/2014 (GBP)	3.5%; 3.5%	Cost per QALY	N/A	N/A
Perri et al. [19]	USA Office setting in rural communities	People with obesity (BMI ≥ 30 kg/m^2^ and ≤ 45 kg/m^2^) 36 kg/m^2^	Intervention groups–Initial weekly sessions (8 for low intensity (Low), 16 for moderate intensity (Mod), and 24 for high intensity (High)), 1200–1800 kcal/day, physical activity advice	Sixteen nutrition education sessions	Lifestyle WMP	RCT (2; N/A)	Unclear	2007 (US$)	NR	Cost per kg lost	N/A	N/A
Rhodes et al. [20]	USA Community	Adults attending African American churches, with BMI ≥ 25 kg/m^2^ and without diabetes ~36 kg/m^2^	Diabetes prevention using Fit Body and Soul (FBAS) intervention: church health advisors provide 12 weekly group sessions and 6 monthly sessions. Included prayer, discussion on scriptures and dietary and physical activity advice (and pedometer), and behavioural modification	Health education by church health advisors	Lifestyle WMP: WMP v education control	RCT (1; N/A)	Unclear	2017 (US$)	N/A	Cost per kg lost Cost per cm reduction in weight circumference	N/A	N/A
Ritzwoller et al. [34]	USA Community centres	BMI 30–50 kg/m^2^ with hypertension 37 kg/m^2^	Community healthy eHealth eating and physical activity advice WMP	Self-help booklet	Remote v control	RCT (2; N/A)	Community health centres	2009 (US$)	NR	Cost per kg lost Cost per unit blood pressure change (mmHg)	N/A	N/A
Tsai et al. [22]	USA Not clear	Women with obesity 38 kg/m^2 c^	Brief Lifestyle Counselling: counselling on calorie restriction and physical activity advice (quarterly provider visits plus monthly weight loss counselling visits) Enhanced Brief Lifestyle Counselling: as above plus choice of meal replacements or weight loss medication	Usual care (quarterly visits with primary care provider)	Lifestyle WMP	RCT (2; N/A)	Health care payer	2011 (US$)	3%; 3%	Cost per QALY	N/A	N/A
Zhang et al. [23••]	USA Primary care	People with BMI > 25 kg/m^2^ (or > 27 kg/m^2^ if receiving insulin therapy) and type 2 diabetes 36 kg/m^2^	Look AHEAD used a low-fat reducing diet, a calorie goal of 1200–1800 kcal/day, initial meal replacements or meal plans, a tailored exercise programme, cognitive–behavioural therapy (CBT), group and individual support and follow-up by telephone or e-mail	Standard diabetes support and education. Three group sessions each year focusing on diet, physical activity, and social support	Lifestyle WMP: Look AHEAD v education control	RCT (9.6; N/A)	Health care system	2012 (US$)	3%; 3%	Cost per QALY	N/A	N/A
**WMP-other **^**d**^												
Finkelstein and Kruger [11]	USA Weight loss clinic/home-setting	People with obesity ~35 kg/m^2 e^	WMPs: Weight Watchers (WMP with weekly in-person or online group meetings), Vtrim (WMP with online group support) WMP with low-calorie meal replacements called Jenny Craig Drug therapies: 120 mg orlistat taken 3 times daily (plus a calorie reduction; most orlistat studies included in their systematic review reported reduction of ~500–900 kcal/day), and Qsymia 7.5 mg phentermine and 45 mg topiramate combination taken once-daily (plus a calorie reduction 500 kcal/day, LEARN manual and monthly visits)	The control arm was a combination of all the control arms of the RCTs included in the systematic review. For WMPs it was usual care, provision of a self-help booklet, or using eDiets (online support of eating habits). For orlistat it was placebo plus the same diet as the intervention group	Lifestyle WMP: Weight Watchers Jenny Craig Vtrim Orlistat	Meta-analysis (min. of 1; extrapolated benefits to 4 years)	Payer	2013 (US$)	N/A; NR	Cost per QALY	N/A	N/A
Finkelstein and Verghese [10]	USA Weight loss clinic/home-setting	People with BMI > 25 kg/m^2^ ~35 kg/m^2 e^	Weight Watchers, 120 mg or 60 mg orlistat 3 times daily and WMP with 500–900 kcal/d deficit versus same WMP and placebo, and a WMP with low-calorie meal replacements called Jenny Craig (1200–2000 kcal/d)	Doing nothing (no intervention cost and no QoL gains because assuming no weight loss)	Lifestyle WMP: Weight Watchers online with and without tracking device v control Weight Watchers in person v control/self-help Orlistat and WMP v WMP and placebo Jenny Craig v control	Meta-analysis (1; extrapolated benefits to 4 years)	Payer	2017/2018 (US$)	N/A; 3.5%	Cost per QALY	N/A	N/A
Krukowski et al. [35]	USA Clinical centres	People with obesity ~36 kg/m^2^	Weekly 1-h online meetings via a synchronous chat group. Calorie restricted diet and dietary fat goal < 25% of calories from fat. Graded exercise goals. Internet condition met weekly in small groups of 15 to 20 individuals in a secure online chat room. Online database to help monitor calorie intake (Calorie King, Family Health Network, Costa Mesa, CA)	Same WMP and weekly one hr face-to-face groups for 6 months	Remote v in-person	RCT (0.5; N/A)	Payer and participant	NR (US$)	N/A	Cost per life year gained	N/A	N/A
Tsai et al. [21]	USA Medical centre	BMI ≥ 35 kg/m^2^ 43 kg/m^2^	< 30 g/day of carbohydrate, no energy reduction goal given	Low-fat reducing diet with energy reduction goal	Lifestyle WMP	RCT (1; N/A)	Society	NR (US$)	N/A	Cost per QALY	N/A	N/A
**WMP-Decision models**												
Avenell et al. [6••] REBALANCE	UK Primary (WMPs) and secondary care (bariatric surgery)	People with severe obesity (BMI of 35 and over) Analysis only included population with BMI ≥ 35 kg/m^2^	Weight management programmes (WMPs) included with data from systematic reviews of RCTs:–WMP1: less intensive diet and physical activity advice, e.g. 12-week commercial weight loss programmes.- WMP2: more intensive lifestyle intervention than WMP1, modelled on shortened Look AHEAD study/US Diabetes Prevention Program (DPP) - VLCD: providing ≤ 800 kcal/day (± 10%), also evaluated addition to WMP1 compared to WMP1 alone.- Look AHEAD: a very long-term intensive diet, exercise and behavioural weight-loss intervention based on the DPP -Bariatric surgery (Roux-en-Y gastric bypass (RYGB) surgery)	Baseline (UK-representative) general population BMI trends	Bariatric surgery, lifestyle WMP	Systematic review and meta-analysis reported in the same NIHR report (4–9; lifetime)	NHS and PSS	2016 (GBP)	1.5%; 1.5%	Cost per QALY	The UK Health Forum microsimulation model	Type 2 diabetes, obesity related cancers, stroke, CHD, hypertension and knee osteoarthritis
Gray [24•]	UK, See Wyke 2015	See Wyke 2015	See Wyke 2015	See Wyke 2015	Lifestyle WMP: WMP v control	RCT (1 (control)-3.5 (intervention); lifetime)	NHS and PSS	2011/2012 from the RCT and 2014/2015 from the follow-up study at 3.5 years (GBP)	3.5%; 3.5%	Cost per QALY	See Wyke 2015	See Wyke 2015
Kent et al. [26•]	UK Primary care	People with BMI > 30 kg/m^2^ 37 kg/m^2^	Primary care referral to a commercial VLCD (Cambridge Weight Plan UK, 810 kcal/d for 8 weeks, and thereafter gradual food reintroduction for 4 weeks, 15 sessions over 24 weeks	Primary care nurse led behavioural support programme by for 12 weeks	Lifestyle WMP: VLCD v low-dose WMP	RCT (1; lifetime)	NHS health care	2016–2017 (GBP)	1.5%; 1.5%	Cost per QALY	Population-based, proportional, multistate life table model. The model links BMI to mortality and noncommunicable disease morbidity (type 2 diabetes, coronary heart disease, stroke, and cancers of the breast, colon, liver, kidney, and pancreas)	N/A
Lewis et al. [27]	England Specialist obesity clinic	BMI ≥ 30 kg/m^2^ with referral 36 kg/m^2^	LighterLife Total is a WMP with a VLCD (600 kcal/d) component and participants are provided with meal replacements, subject to behavioural change therapy and group support	No treatment, Counterweight, Weight Watchers, Slimming World, GB and GBP	Lifestyle WMP	RCT (3; 10)	NHS England	2012 (GBP)	3.5%; 3.5%	Cost per QALY	Economic model not described	NR
Meads et al. [28]	UK Primary care, community	People with obesity 35 kg/m^2^	Referral by a health professional in primary care to a commercial WMP group (Slimming World) for usually 12 weeks	Information provision either verbally or printed material only	Lifestyle WMP	RCT (1; lifetime)	Personal health and social services	NR (GBP)	3.5%; 3.5%	Cost per QALY	Markov model	T2DM, primary stroke, primary MI, T2DM + stroke, T2DM + MI, secondary stroke, secondary MI
Miners et al. [36]	UK Remote (communication technology)	People with obesity (BMI ≥ 30 kg/m^2^) 35 kg/m^2^ (subgroup analysis)	An e-learning device (website) provided advice, tools and information to support behaviour change in terms of dietary and physical activity patterns, as required. Personalised motivational statements were provided, based on online questions E-mail reminders were sent if individuals who had not been active on the website	Conventional care: including physical activity and/or dietary advice (excluding e-learning devices or pharmacological treatment)	Remote v control	Systematic review and meta-analysis (not specified; lifetime)	UK health services	2009 (GBP)	3.5%; 3.5%	Cost per QALY	Discrete event simulation model	Disease state (CVD fatal event, CVD, non-fatal event, survivor of CVD, T2D)
Nuijten et al. [29]	USA Secondary care	People without type 2 diabetes and with BMI 30–39.9 kg/m^2^ (starting BMI = 35 kg/m^2^) Short term: effectiveness based on study with mean BMI = 43 kg/m^2^ Long-term: effectiveness data based on study with mean BMI = 40.8 kg/m^2^	OPTIFAST programme: ≤ 800 kcal/d VLCD for 12 weeks, gradual food reintroduction, followed by maintenance phase up to 52 weeks with nutrition education, physical activity, behavioural training	No intervention (constant BMI = 35 kg/m^2^)	Lifestyle WMP: VLCD v control Also: VLCD v surgery	RCT (1; 3)	Healthcare payer	2016 (US$)	5%; 5%	Cost per QALY	Decision tree	Class I and II obesity (with or without T2DM), class III obesity, BMI reduction/no reduction, maintain BMI reduction/regain weight post BMI reduction, have increased risk of obesity complications if no BMI reduction/regain weight
Radcliff et al. [30]	USA Community	People with BMI 30–45 kg/m^2^ 36 kg/m^2^	There were three intervention groups (with different intensities) given regular sessions based on DPP: Low (8 nutrition education sessions), Mod (16 nutrition education sessions) and High (24 nutrition education sessions) behavioural coaching giving dietary advice (1200–1800 kcal/d) and physical activity advice	Eight nutrition education sessions	Lifestyle WMP: Low-dose WMP v education control Moderate intense WMP v education control High intense WMP v education control Moderate intense WMP v education control	RCT (2; 5)	Third party payer	2015 (US$)	3%; 3%	Cost per QALY	Patient level Markov model	Normal blood sugar, Pre-diabetes, Type 2 diabetes
Thomas et al. [31]	UK Primary care	People at high risk of diabetes BMI ≥ 35 kg/ m^2^ subgroup mean BMI not reported)	NHS Diabetes Prevention Programme (DPP): intensive lifestyle management programme with dietary and physical activity advice	No diabetes prevention intervention (baseline England representative population cohort)	Lifestyle WMP: WMP v control	Systematic review and meta-analysis commissioned by PHE (1–4.2; 20)	NHS	Unclear (GBP)	3.5%; 1.5%	Cost per QALY	Individual patient simulation model	Diabetes, hypertension, cholesterol, CVD, cancer, osteoarthritis, depression
Trueman et al. [32]	UK GP practices	People with obesity 37 kg/m^2^	Counterweight delivered by a practice nurse in groups or individual sessions (nine over 12 months). Patients chose either a goal-setting approach or were prescribed a calorie deficit (≥ 500 kcal/day)	No treatment; followed an expected trajectory (broadly representative of the UK population) without the Counterweight intervention	Lifestyle WMP	Prospective cohort study (2; 10)	Health care payer	2005 (US$)	3.5%; 3.5%	Cost per QALY	Individual-level simulation model	Gain weight, lose weight, weight unchanged, no weight-related comorbidities, develops diabetes, develops colon cancer, develops CHD
Wilson et al. [ 40]	USA Community centre	Low-socioeconomic-status Mexican-origin ≥ 40 kg/m^2 ^subgroup mean BMI not reported)	12-week community-based WMP (called Beyond Sabor) with a physical activity programme Weekly 2-h classes which included physical activity, and education (including cooking demonstration and group interaction) to promote a healthy diet	Usual care, not clearly described	Lifestyle WMP	Not specified (not specified, 20) ^f^	Participant	NR (US$)	3%; NR	Cost per QALY	Estimated outcomes using the Archimedes Outcomes Analyzer	NR
Wyke et al. [25]	UK Football clubs	Men with BMI ≥ 28 kg/m^2^ 35 kg/m^2^	FFIT Group: The FFIT had pitch-side physical activity sessions led by club community coaching staff and an incremental pedometer-based walking programme. The dietary component of FFIT was designed to deliver a 600-kcal/day deficit	Given a booklet on losing weight. Waiting list (could do the programme 12 months later)	Lifestyle WMP	RCT (1; lifetime)	NHS	Unclear, seems to be 2011/12 (GBP)	3.5%; 3.5%	Cost per QALY	State transition model/risk factor model	CVD event-free, non-fatal CHD, non-fatal CBVD, fatal CVD and fatal non-CVD
**Pharmacotherapy studies–decision models**^**g**^												
Hertzman [38]	Sweden Clinics	People with obesity 36 kg/m^2^	120 mg orlistat (up to 3 times/day) in addition to a low-fat diet with calorie reduction for 12 months	Placebo plus a low-fat diet with calorie reduction	Orlistat	5 RCTs pooled (1; different for costs and outcomes)	Swedish healthcare system	2003 (Euro)	3%; 3%	Cost per QALY	Decision tree	Responders (continue Orlistat), non-responders (discontinue Orlistat), T2DM, no T2DM
Lacey et al. [39]	Ireland Weight loss clinics	People with severe obesity 36 kg/m^2^	Orlistat (majority of studies were orlistat with the dose 120 mg 3 times daily) and low-fat calorie reduced diet	Placebo and low-fat calorie reduced diet	Orlistat	5 RCTs (1; different for costs and outcomes)	Irish health-care perspective	2003 (Euro)	3%; 3%	Cost per QALY	NR	NR
Veerman et al. [37]	Australia Primary care	People with obesity 37 kg/m^2^	120 mg orlistat 3 times daily for 12 months and (on average) 1.6 medication-related follow-up visits per person to the GP	Australian reference population based on existing levels of morbidity and mortality for 2003	Orlistat	Meta-analysis study (1; lifetime)	Both health sector perspective and patient perspective	2003 (AU$)	3%; 3%	Cost per DALY	Proportional multi-state life table Markov model	Stroke, ischemic heart disease, hypertensive heart disease, T2DM, osteoarthritis, post-menopausal breast cancer, colon cancer, endometrial cancer and kidney cancer

*BMI* body mass index, *DALY* disability adjusted life year, *DASH* Dietary Approaches to Stop Hypertension, *FFIT* Football Fans in Training, *GB* gastric banding, *GPB* gastric bypass, *Look AHEAD* Look Action for Health in Diabetes, *NHS* National Health Services, *N/A* not applicable, *NR* not reported, *Mod* moderate, *PSSRU* Personal Social Services Research Unit, *QALY* quality adjusted life year, *RCT* randomised controlled trial, *T2DM* type 2 diabetes mellitus, *VLCD* very low-calorie diet, *WMP* weight management programme

^a^The Dead state was always included in the health state decision models

^b^Note, trial follow-up in the RCT that the economic evaluation is based on is 36 months (with the intervention stopping at 24 months, based on the Look AHEAD lifestyle intervention)

^c^Average BMI calculated by authors using study data

^d^Other study design is used to describe studies that are classified as neither RCT-based economic evaluations nor decision analysis models

^e^Mean BMI not reported in paper. Calculated from supplementary table with mean BMI from each group

^f^Where primary treatment effectiveness sources is marked as “not specified”, this means it was not possible to directly identify the source of treatment effectiveness (i.e. weight loss) data used for the economic evaluation

^g^Note, Finkelstein 2014 and Finkelstein 2019 evaluated multiple interventions, one of them was also Orlistat. That gives a total of 5 studies evaluating Orlistat

### Cost-Effectiveness Results

**Fig. 2:**
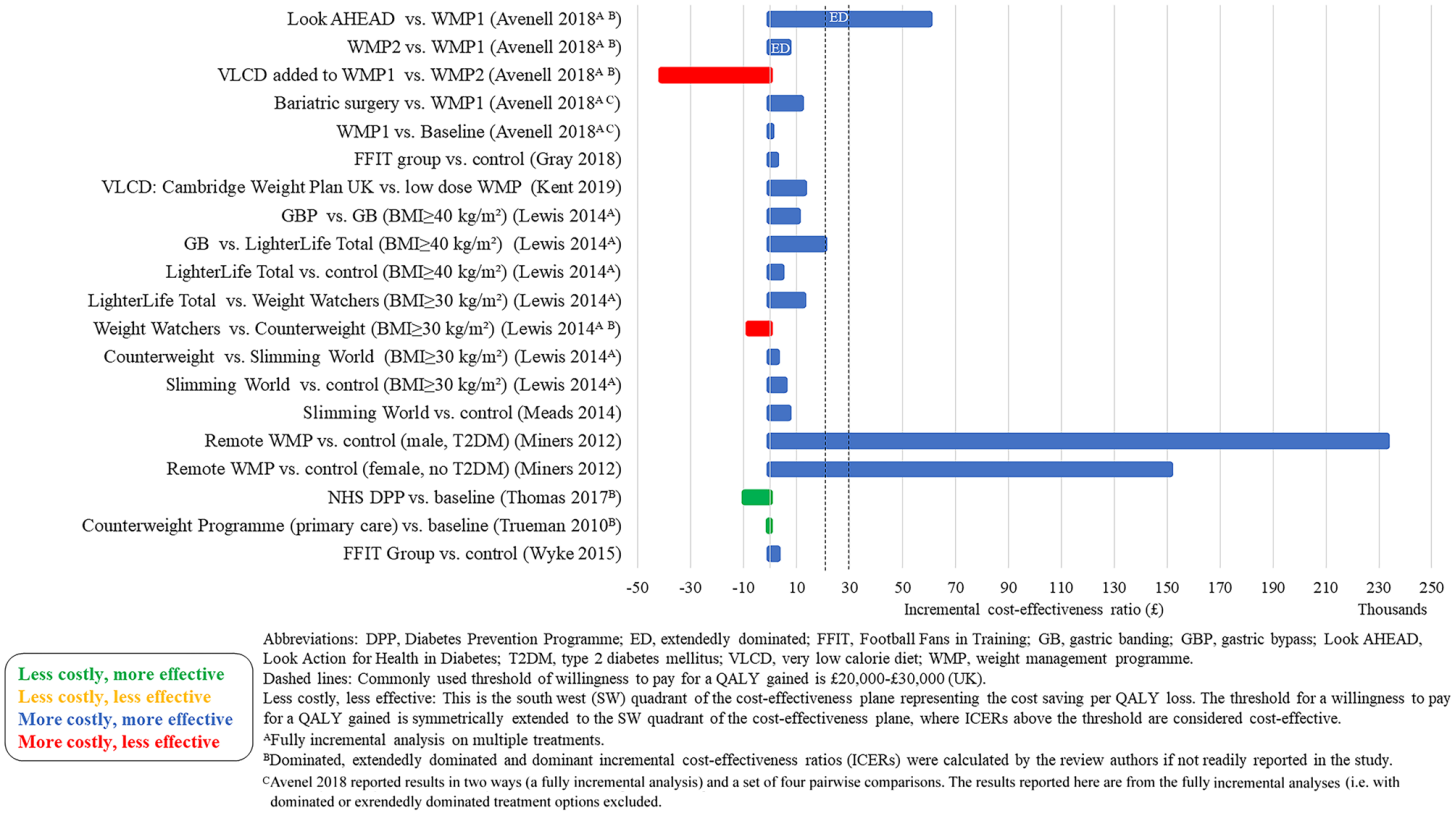
Cost-effectiveness results–weight management programmes–decision models (cost per QALY (£))

**Fig. 3:**
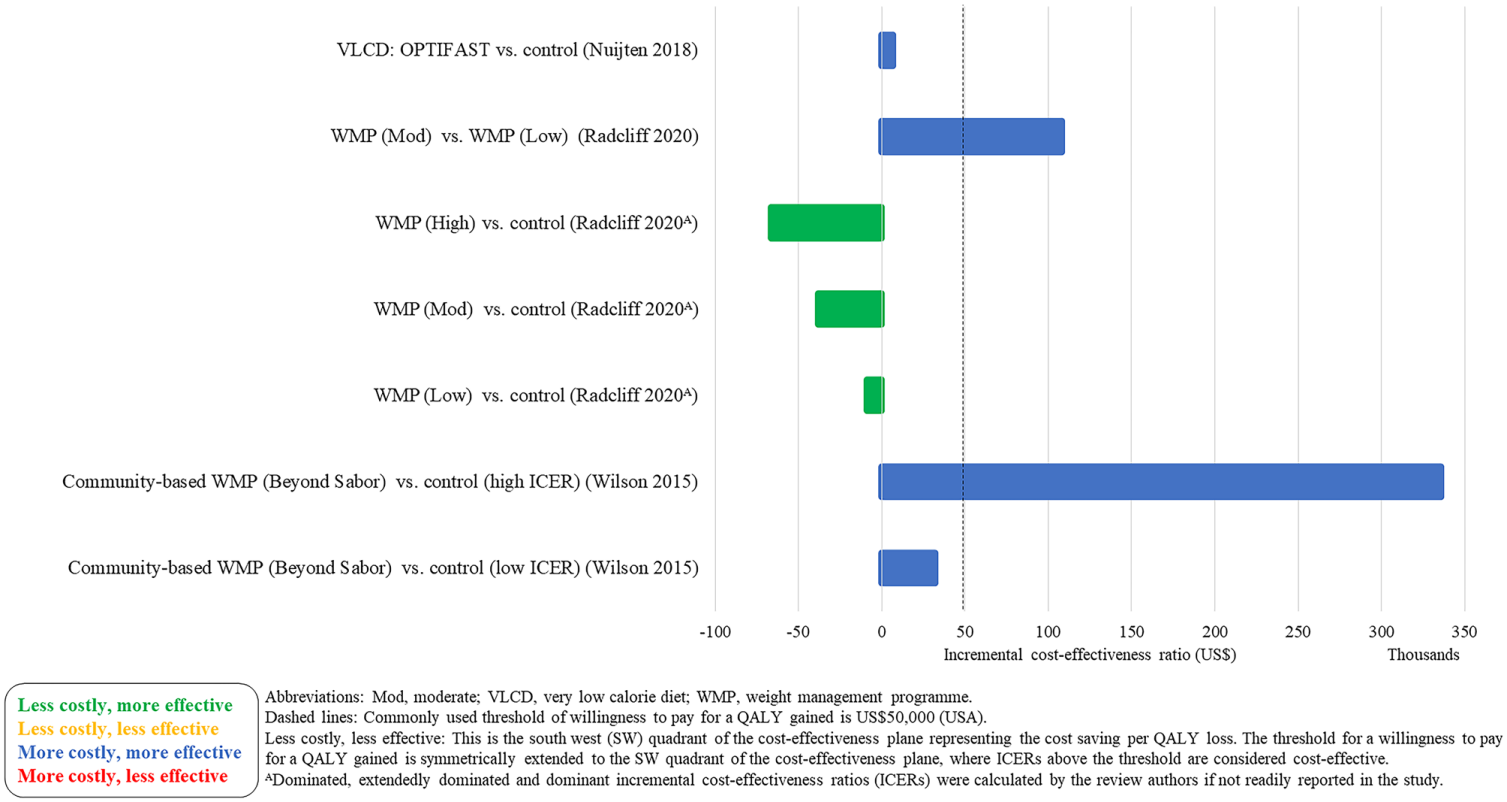
Cost-effectiveness results–weight management programmes–decision models (cost per QALY (US$))

**Fig. 4:**
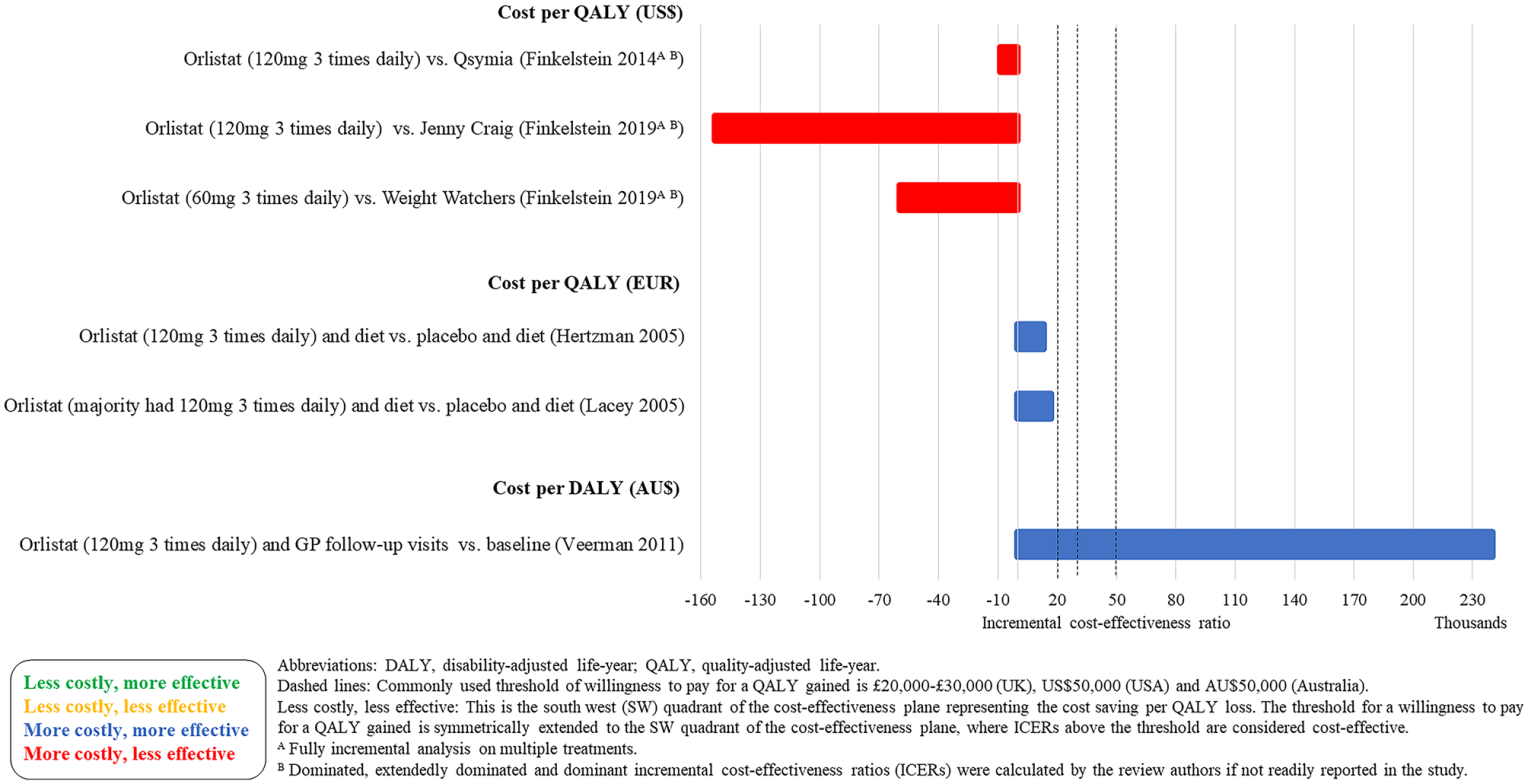
Cost-effectiveness results–pharmacotherapy–decision models (cost per QALY (EUR) and cost per DALY (AU$))

**Fig. 5:**
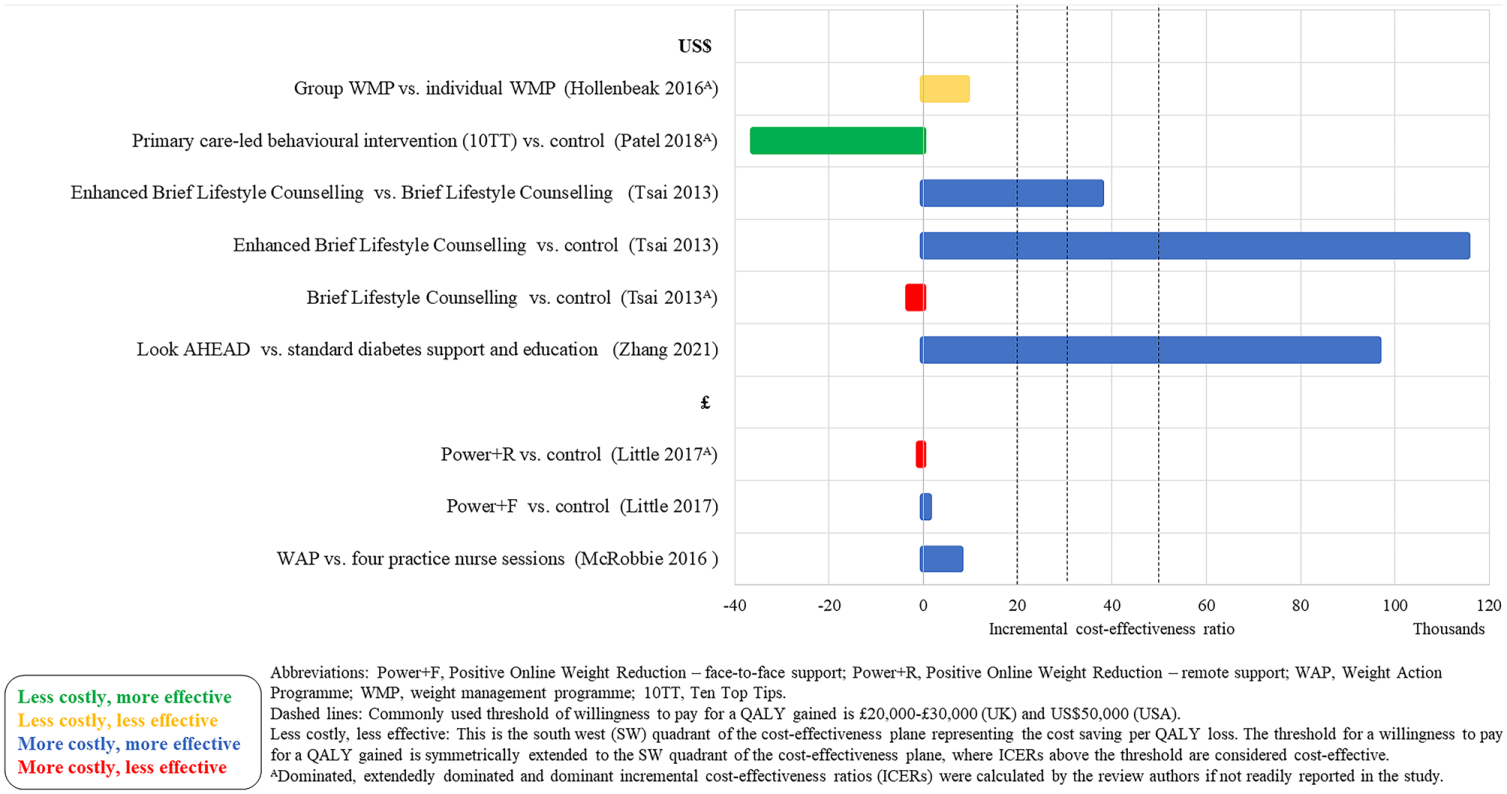
Cost-effectiveness results–weight management programmes–within trial economic evaluations (cost per QALY (US$, £))

**Fig. 6:**
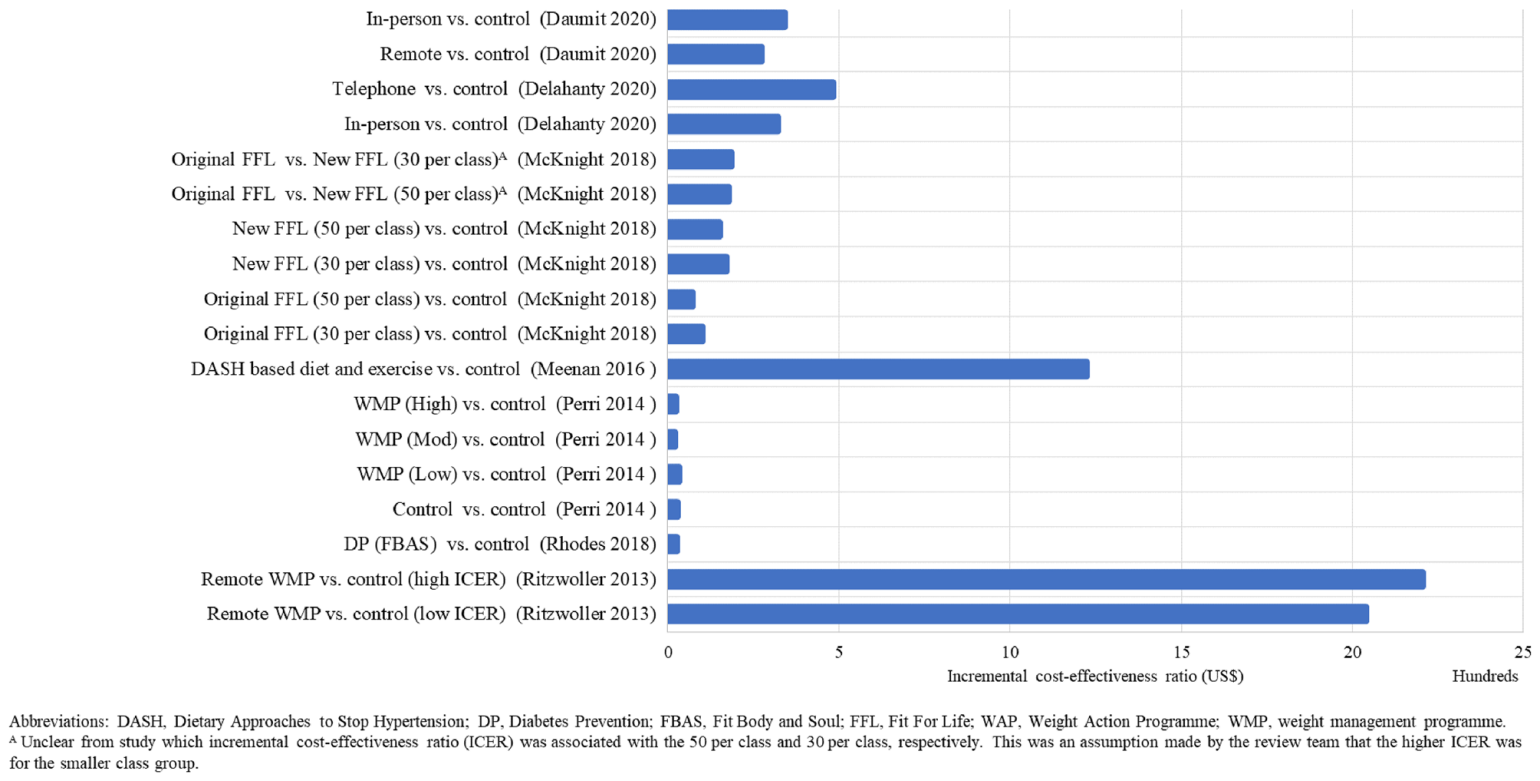
Cost-effectiveness results–weight management programmes–within trial economic evaluations (cost per kg lost (US$))

**Fig. 7:**
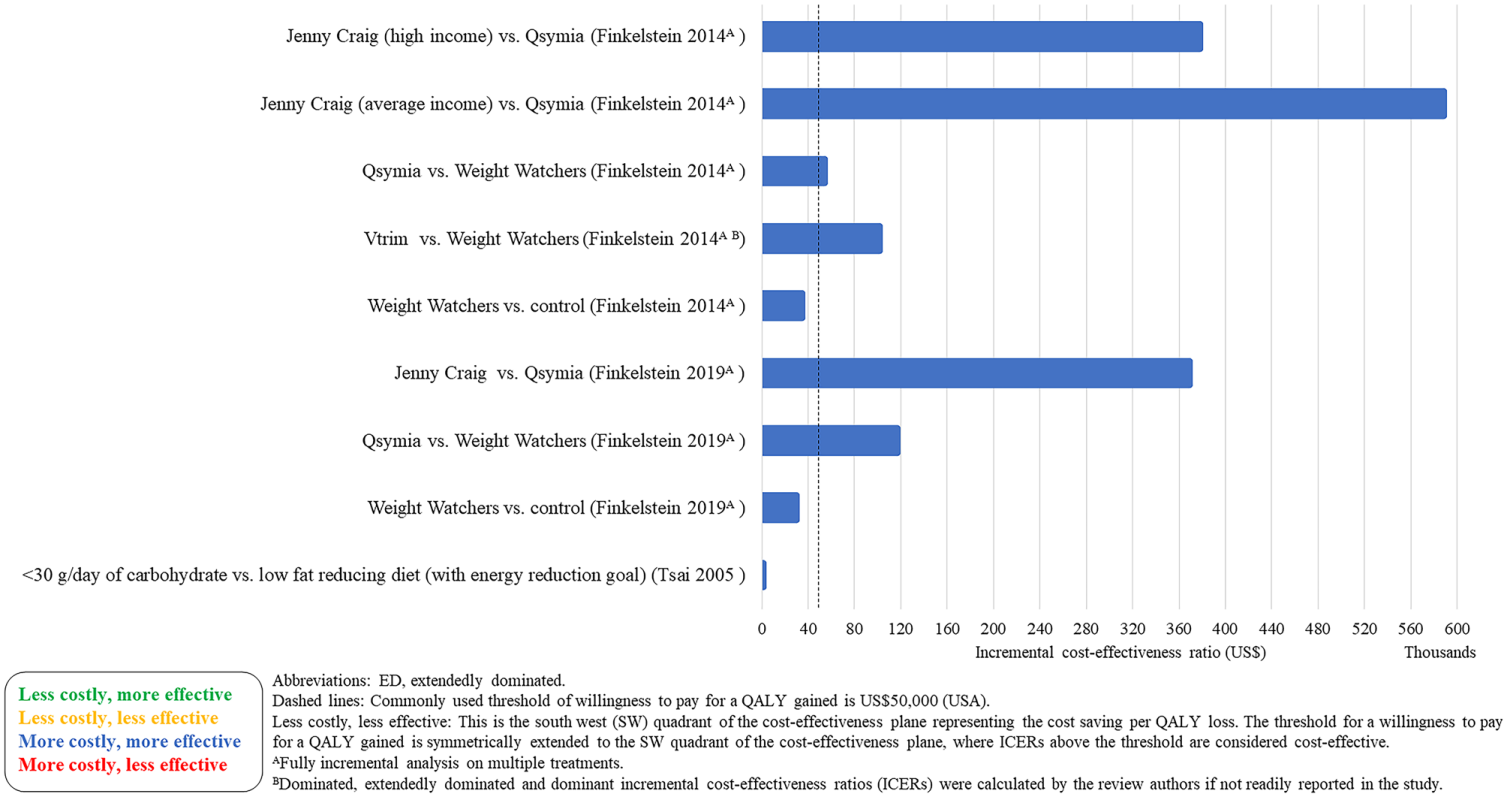
Cost-effectiveness results–weight management programmes–neither within trial economic evaluations nor decision models (cost per QALY (US$))

The cost-effectiveness results are presented in Figs. 2, 3, 4, 5, 6, and 7. The control groups are described in detail in Table 1 and include a variety of minimal interventions such as do-nothing, self-help booklet and usual care. More detailed results are reported in the Supplementary Material Table 2. A summary of results for each WMP category is provided below.

#### Weight Management Programmes (WMP)

Lifestyle WMPs (11 within trial, 11 decision models and 3 neither within trial nor decision models) included diet and physical activity advice [6••, 12, 13, 15–22, 24•, 25, 30, 31, 40], low carbohydrate diets [14, 21], commercial WMPs (Weight Watchers and Vtrim, Slimming World) [10, 11, 28, 32], the Counterweight programme [19] and Look AHEAD [6••, 23••]. The comparators were either no active treatment (most often occurring in decision models) or usual care, with heterogeneous definition of usual care across the studies. Many studies include a “usual care” comparison arm that includes an active intervention/education that may not necessarily reflect usual care as delivered to the general population. The duration of follow-up varied from 12 weeks to 9.6 (median) years, with the majority of studies having a follow-up of 1–2 years. The longest follow-up intervention was Look AHEAD. The ICERs across studies ranged from: US$22 to US$1224 per kg lost for CEAs and from dominant (i.e. less costly and less effective vs different dietary advice) to US$335,952 (vs unclearly described usual care) per QALY for CUAs. The ICER for the WMP with the longest follow-up (Look AHEAD) was uncertain in the within trial analysis [23••] and borderline cost-effective (vs baseline population trends) or extendedly dominated (vs other non-surgical and surgical WMPs) [6••].

Four studies [6••, 26•, 27, 29] (all decision models) included a VLCD as an intervention [6••, 26•, 27, 29]. The VLCD interventions (LighterLife Total [27], Optifast [29], Cambridge Weight Plan UK [26•] and different meta-analysed VLCD interventions [6••]) were followed by a WMP of varying intensity. Duration of follow-up varied from 1 to 4 years across the VLCD studies. The ICERs for the VLCD intervention ranged from US$6,475 (vs no intervention) per QALY [29] to dominated (i.e. more costly and less effective compared to other WMPs and bariatric surgery) [6••].

Two meal replacement studies [10, 11] were included (neither of which were within trial nor decision model but extrapolated benefits using meta-analysed data). In both studies, the Jenny Craig meal replacement intervention included a prescribed calorie intake and counselling. Jenny Craig was compared to other WMPs, with ICERs ranging from to US$369,000 [10] to US$588,620 per QALY [11].

A group intervention (within trial) included counselling through a conference call, instead of individually (control group) [33]. The ICER was US$9249 (less costly, less effective). Follow-up was only 1 year.

The interventions that were delivered remotely (4 within trial, 1 decision model and 1 neither within trial nor decision model) were Internet or telephone-based. Other evaluations were for interventions delivered remotely rather than in-person [12–14, 35, 36]. Follow-up ranged from 6 months to 2 years. The ICER ranged from US$275 [12] to US$2204 [34] per kg lost for CEAs and £151,142 to £232,911 (vs usual primary care; the decision modelling study) per QALY [36] for CUAs.

Five studies (3 decision models and 2 neither within trial nor decision model) evaluated the cost-effectiveness of Orlistat and low-fat diet and showed mixed results [10, 11, 37–39]. When compared to placebo (plus a low-fat diet), Orlistat was cost-effective [38, 39]. However, when compared to existing population trends or more intense interventions (that were defined as usual care), Orlistat was no longer cost-effective [10, 11, 37]. Orlistat was not cost-effective in the lifetime decision modelling study [37].

Some interventions were evaluated in multiple studies. Counterweight was deemed cost-effective when compared to no treatment [32]. However, Counterweight was not cost-effective compared to Weight Watchers [27]. Slimming World was cost-effective compared to being given information verbally or through written material [28]. However, in a different study, Slimming World was not found cost-effective compared to Counterweight, Weight Watchers and Lighterlife Total [27]. Look AHEAD was borderline cost-effective compared to baseline population trends [6••] but mixed results when compared to a lifestyle WMP including physical activity and dietary advice [6••, 23••].

The majority of studies were conducted in the USA (*n* = 17). The WMPs considered cost-effective in the longer term (in terms of cost per QALY) in the USA were OPTIFAST (a VLCD) [29] (but with a 3-year time horizon) and a lifestyle intervention based on DPP [30] (but with a 5-year time horizon). The WMPs that were considered cost-effective in a UK setting (*n* = 12) in the longer term were the WMP delivered in a football club [24•, 25], Lighterlife Total [27], Slimming World (only when compared to usual care) [28], the Counterweight Programme (only when compared to no treatment) [32], Cambridge Weight Plan [26•] and NHS Diabetes Prevention Programme [31]. The WMP considered in Sweden (*n* = 1), Ireland (*n* = 1) and Australia (*n* = 1) was Orlistat, with ICERs ranging from €13,125 per QALY (vs placebo plus a low-fat diet) [38] to dominated (vs more intense interventions) [10, 11].

Note that all the cost-effectiveness results here are compared against different thresholds, with differing health care systems and methodological quality. Therefore, in the following section, we will assess the methodological quality of the studies.

## Quality Assessment

### Trial-Based Economic Evaluations

About half of the economic evaluations were trial-based. The follow-up period for most studies ranged between 1 and 2 years. Studies with longer (than 2 years) follow-up periods were 3.5 years [24•], 5 years [6••] and about 9 years (Look AHEAD). Within trial, economic evaluations do not capture the long-term costs and benefits, nor assumptions associated with a treatment for severe obesity due to the long-term impact on ORDs.

### Decision Models

The following sections reflect the key methodological issues identified in the quality assessment of the included modelling studies. The most common model types were a Markov model and individual level simulation/microsimulation model. The most common framework for analysis was CUA, and the most common benefit measurement was the quality adjusted life year (QALY). The incremental cost effectiveness ratio (ICER) was therefore compared to a commonly used country-specific threshold.

#### Model Structure

Decision model time horizons ranged from 3 years to a lifetime horizon across the studies. 8/15 (53%) of decision models were built on a life-time horizon, which is likely required to capture all the costs and consequences of ORD such as stroke, cancer, diabetes and myocardial infarction. The varying time horizons further limit the comparability between the studies. Short-term decision models, such as those conducted over only 3 years are insufficient for decision making as they fail to capture the long-term benefits of weight loss interventions on ORD and may generate cost-effectiveness conclusions biased against WMPs. However, a counterargument is that longer term extrapolations require assumptions about the impact of transient weight loss on ORD, and assumptions about the long-term rate of weight regain over time (Weight Regain Assumptions). Longer term extrapolations, based on short-term data, add uncertainty to results, with a risk of drawing cost-effectiveness conclusions that are biased towards WMPs. To determine the most likely cost-effectiveness conclusions from a decision model, it is critical that models include a comprehensive range of sensitivity analyses to ascertain the impact of important assumptions such as transient effects and weight regain rates on results.

Furthermore, many of the obesity models did not include many of the relevant disease health states such as T2DM, stroke, cardiovascular disease, and obesity-related cancers. Some obesity models [6••, 24•, 26•, 31] (all UK studies) did include many of the ORD risks factors such as T2DM (all studies), obesity-related cancers [6••, 26•, 31], stroke [6••, 24•, 26•], coronary heart disease [6••, 24•], hypertension [6••, 24•, 31], knee osteoarthritis [6••, 31] and congestive heart failure [31]. Obesity-related cancers included breast, colon, liver, kidney and pancreas cancers. The populations considered in the decision models were a mixture of the general population with obesity, with T2DM, at high risk of T2DM or with comorbidities. Two decision models only focused on T2DM [30, 38]. Whilst this is suitable for studies only interested in T2DM as an outcome, the exclusion of other health states from studies modelling interventions for severe obesity may tend to underestimate the benefits of weight loss interventions in the long-term.

#### Weight Regain Assumptions

The modelling assumption on weight regain over time varied widely between the studies. This parameter is subject to uncertainty as we do not know what happens beyond the short trial time period, which was the case for studies on WMPs.

Studies assumed a variety of weight regain assumptions after the end of intervention delivery. 9/15 (60%) assumed a constant weight regain rate to baseline (often at 1-kg regain per year or a 5-year regain to baseline weight) or a linear projection of the BMI based on trial data. For the remainder of the studies, it was either unclear, not reported or done differently (i.e. assumed QALY gains from weight loss linearly reduced to zero or extrapolated a person’s measured glycated haemoglobin values instead of their BMI).

The weight regain rate has important implications for cost-effectiveness, particularly in models where the risk of ORD is directly linked to time-specific weight/BMI. Long-term follow-up data on WMPs is frequently lacking and therefore exploring the impact that the weight regain assumption has on results is crucially important. The longest follow-up for WMPs identified in the REBALANCE clinical effectiveness review [6••] was from the Look AHEAD study [41], with 9 years of data. This was an intensive longer term WMP which is dissimilar to the other WMPs identified in this review, which had much shorter follow-up. The Look AHEAD study was evaluated in two studies included in this review, one trial-based economic evaluation [23••] and in one decision model [6••]. However, for the majority of WMPs, there is an urgent need for longer term follow-up of RCT evidence to determine the most accurate assumptions for economic modelling.

#### Variation in Interventions and Comparators

The comparisons identified in this review varied widely. The interventions and comparators differed both between WMP categories and within categories. Lifestyle interventions varied widely and were compared to no active treatment (e.g. country-specific population BMI trajectory) or some form of usual care. VLCDs were compared to WMPs with varying intensity. The meal replacement (Jenny Craig) was compared to different WMPs. The group and remote interventions were compared to in-person lifestyle interventions. Because of the variation in the intervention and comparators, it is difficult to compare across the studies.

#### Sensitivity Analyses

Sensitivity analyses are key to unravelling the uncertainty in the cost-effectiveness results. Four studies varied the discount rate [6••, 26•, 28, 36], which generally had negligible impact on the cost-effectiveness results. Only a few studies looked at varying the time horizon, and not surprisingly, the longer the time horizon, the more cost-effective the intervention [6••, 29]. This is because costs are often incurred upfront but the benefits in terms of ORD avoided often occur far into the future.

The weight regain rate was varied in 4 studies [6••, 24•, 26•, 28]. In two of the studies where the weight regain rate was assumed to be more conservative (quicker weight regain to baseline weight) [24•, 28], it did not change the cost-effectiveness conclusions. In one study, the intervention was more cost-effective when assuming a weight that was 1 kg below baseline weight beyond 5 years, rather than assuming that all weight was regained after 5 years. The intervention would remain cost-effective as long as the weight is kept off and is not all regained for at least 3 years [26•]. Lastly, in our REBALANCE study [6••], the weight regain was assumed to follow a linear trajectory based on trial data instead of a 5-year weight regain. Look AHEAD went from being borderline cost-effective to cost-effective (vs baseline population trends) but for the other WMPs evaluated it both increased costs and reduced QALY gains (although remained cost-effective compared to baseline population trends) [6••].

In the younger age group (aged 20–34), a total diet replacement programme [26•] (assuming a 5-year weight regain) was not cost-effective, and the cost per QALY was highest in the older age groups. However, this was not the case when assuming that 1-kg weight loss is maintained beyond 5 years (in this case the intervention was cost-effective for all age groups). This further highlights the importance of varying the weight regain assumption.

For the higher BMI groups, the cost per QALY was lower (still cost-effective in all age groups) [26•] and more cost saving [29].

Only three studies [24•, 25, 36] conducted a value of information analysis (VOI). VOI is a framework for identifying where the greatest uncertainty lies to which future research should be directed. Considering the uncertain longer term weight loss, weight loss maintenance and associated clinical event management, VOI could help guide the direction of future research in the area of obesity.

## Discussion

We identified 32 studies (across 36 papers) evaluating the cost-effectiveness of non-surgical interventions for severe obesity (BMI ≥ 35 kg/m^2^). The cost-effectiveness findings from the WMP and pharmacotherapy studies were mixed. Half of the WMP studies were economic evaluations alongside RCTs, not extrapolating costs and benefits over a longer time horizon, failing to capture the long-term impact of an intervention on obesity, a chronic disease. Furthermore, studies were subject to heterogeneity with regard to the chosen comparators, study populations, settings, decision model structure, costing methodology, weight regain assumptions and time horizons. To our knowledge, this (both our REBALANCE review and updated review) is the first systematic review of economic evaluations of different WMPs for severe obesity (BMI ≥ 35 kg/m^2^).

Two reviews have recently been conducted on the cost-effectiveness of interventions for people with obesity [42, 43]. However, unlike our review, they focused on bariatric surgery only their population of interest was people with obesity (BMI ≥ 30 kg/m^2^) rather than severe obesity (BMI ≥ 35 kg/m^2^), included partial economic evaluations (e.g. cost only, studies or effectiveness evaluations) in addition to full economic evaluations. As in the REBALANCE study, they also found surgery to be cost-effective. One of their included studies [44] applied a post-surgery complication risk over a 10-year period. This is a step in the right direction considering the evidence showing a longer term risk of complications following bariatric surgery [45, 46]. More recent relevant data on longer term surgery complications would improve future obesity decision models.

The quality of the included studies varied. However, as we have learnt from the REBALANCE study, many of these quality assessment items were not captured in the quality assessment checklists. These additional items for the quality assessment checklists would improve the quality assessment of obesity models [7]. Firstly, weight regain assumptions in the decision models varied widely, were poorly justified and were rarely explored in sensitivity analyses (only in 4 studies). This is important especially for WMPs because the majority of WMPs were of short duration and therefore, the longer term weight regain rate is unknown. The assumed weight regain rate (BMI trajectory over time) is associated with an increased risk of developing ORDs. Therefore, an intervention assuming patients revert back to baseline in 5 years’ time is more likely to be cost-effective than assuming patients revert back to baseline BMI immediately. Secondly, many studies did not include all the relevant disease health states such as T2DM and stroke. Lastly, the trial results should be extrapolated over a longer time horizon. Including these items on the quality assessment checklist would be helpful to reviewers in assessing the quality of obesity models.

Two studies in the review (UK studies) evaluated multiple WMPs and bariatric surgery, however, one with only a 10-year time horizon for costs and outcomes [27] and the other with a lifetime horizon for costs and outcomes [6••]. The REBALANCE study [6••] included all the relevant comparators (both surgical and non-surgical options) that were identified through a systematic review of RCTs, and modelled over a lifetime horizon. From a UK NHS perspective, the generalisability of the results in the systematic review presented here to a UK setting is poor. A recent UK RCT was published evaluating a VLCD (DROPLET trial) offered in primary care, and was found to be cost-effective over a lifetime horizon [26•]. However, the only comparator was nurse-led support. There is a need for a comparison of commonly available treatments in the UK NHS.

### Strengths and Limitations

Key strengths of this study are the systematic approach to the literature review in identifying the cost-effectiveness evidence on interventions for severe obesity and the methodological quality assessment of the included studies. Furthermore, this review brings focus to the population with severe obesity, identifying value for money interventions for treating severe obesity.

Due to study heterogeneity, no quantitative synthesis of the study results by meta-analysis was attempted, a common issue with systematic reviews of economic evaluations. This is because studies were conducted in different countries with different health care systems, different definitions of comparator groups, model structures, costing methods and modelling assumptions. A detailed quality assessment was not conducted for all included studies, only for those identified through the REBALANCE review, but this informed our subsequent assessment of studies.

## Conclusions

Most WMPs were cost-effective and pharmacotherapies showed mixed results. However, the cost-effectiveness evidence should be read with caution due to the varying methodological issues and study heterogeneity across the studies. About half of the WMPs were economic evaluations alongside RCTs, not accounting for the difference in long-term costs and outcomes between the considered interventions, crucial for a chronic disease such as obesity. WMPs tended to have short-term follow-up, rendering it even more important to make use of decision models. Decision models did not include most relevant health states and had varying assumptions around weight regain which was rarely explored in sensitivity analysis.

Although there exists a decision model assessing different types of interventions [6••], there is still a need for future economic evaluations to focus on effective interventions available on the UK NHS for people with severe obesity. Furthermore, there is room for improvement with regard to obesity models and their methodology. To improve decision models, there is a need for the inclusion of all the important health states, improved consistency in the assumed weight regain rate (which ideally should be based on best available evidence), and improved transparency in the description of the comparators (and interventions) to allow better comparison across studies.

## Supplementary Information

Below is the link to the electronic supplementary material.

Supplementary file1 (DOCX 25 KB)
