# Supplementary material for: A Systematic Review of the Evidence for Non-surgical Weight Management for Adults with Severe Obesity: What is Cost Effective and What are the Implications for the Design of Health Services?
Source: Curr Obes Rep. 2022 Nov 21;11(4):356–85. doi: 10.1007/s13679-022-00483-z (PMC9729129; doi:10.1007/s13679-022-00483-z)
Supplement: Supplementary file 1 — Supplementary file1 (DOCX 25 KB) [file 13679_2022_483_MOESM1_ESM.docx]

**Supplementary Material**

**Table 1 Data extraction template**

| **Study characteristics** | Description of intervention |
| --- | --- |
|  | Description of control |
|  | Intervention type |
|  | Country |
|  | Setting |
|  | Population |
|  | Primary treatment effectiveness source |
|  | Trial duration |
| **Economic characteristics** | Weight regain assumption |
|  | Economic evaluation type |
|  | Benefit measure |
|  | Study type |
|  | Primary economic outcome measure |
|  | Costing perspective |
|  | Cost year |
|  | Currency |
|  | Discount rate for costs and benefits |
|  | Model description |
|  | Health states model (description of health states) |
|  | Base case time horizon |
|  | Whether probabilistic sensitivity analysis was conducted |
|  | Whether value of information analysis was conducted |
| **Cost-effectiveness results** | Incremental cost effectiveness ratio |
|  | Assessment of uncertainty (probability of cost-effectiveness) |
|  | Threshold used |
|  | Which sensitivity analysis was conducted |

**Table 2 Cost-effectiveness results**

| **Study** | **EE Type** | **Benefit measurement** | **Model base-case time horizon** | **PSA?** | **ICER^A^** | **Assessment of uncertainty (probability of cost-effectiveness)** | **Threshold** |
| --- | --- | --- | --- | --- | --- | --- | --- |
| **WMP – economic evaluation alongside RCT** | | | | | | | |
| Daumit 2020 | CEA | kg lost | -- | -- | Control: N/A (cost assumed to be minimal and therefore zero); In-person: US$342/kg lost Remote: US$275/kg lost | NR | NR^B^ |
| Delahanty 2020 | CEA | kg lost & 1%/5% weight lost | -- | -- | Cost per kg lost: In-person vs usual care: US$321;  Telephone vs usual care: US$483  Cost per 1% weight lost: In-person vs usual care: US$296;  Telephone vs usual care: US$432  Cost per 5% weight lost: In-person vs usual care: US$2960;  Telephone vs usual care: US$5613 | Cost per kg lost ^C^: In-person/telephone vs usual care: 0-100% (US$0-US$5000) Telephone vs in-person: 0-~50% (US$0-US$5000) Cost per additional person with 5% weight lost: In-person vs usual care: 0-100% (US$0-US$10000) Telephone vs usual care: 0-~85% (US$0-US$10000) Telephone vs in-person: 0-~17% (US$0-US$10000) | NR^B^ |
| Hollenbeak 2016 | CEA/CUA | QALY | -- | -- | US$9249 (intervention is less costly and less effective) | 48% | US$100,000 |
| Little 2017 | CEA/CUA | QALY | -- | -- | Power+F vs. control: £1203;^D^  Power+R vs. control: -£966^D^ | NR | NR |
| McKnight 2018 | CEA | kg lost | -- | -- | Original FFL diabetes prevention programme: US$101 (30 per class);  US$73 (50 per class)  New FFL diabetes prevention programme: US$170 (30 per class);  US$151 (50 per class)  ICER comparing Original FFL to new FFL : US$178-184 (depending on class size 30/50). | NR | NR |
| McRobbie 2016 | CUA | QALY | -- | -- | £7,742 | 68.26%  77.46% | £20,000 £30,000 |
| Meenan 2016 | CEA | kg lost | -- | -- | US$1224 | NR | NR |
| Patel 2018 | CUA | QALY | -- | -- | Dominant | 52% (@ both £20k and £30k) | £20,000-£30,000 |
| Perri 2014 | CEA | kg lost | -- | -- | Control: US$28;  Low: US$33;  Mod: US$22;  High: US$25 | NR | NR |
| Rhodes 2018 | CEA | kg lost and reduction in waist circumference | -- | -- | US$26.52 per kg of weight lost  US$21.00 per one cm reduction (in waist circumference) | NR | NR |
| Ritzwoller 2013 | CEA | kg lost & blood pressure change (mmHg) | -- | -- | Ranged from US$2040 to US$2204 per kg lost  Ranged from US$574 to US$621 (per mmHg) | NR | NR |
| Tsai 2013 | CEA/CUA | QALY | -- | -- | Brief Lifestyle Counselling vs. usual care: dominated;  Enhanced Brief Lifestyle Counselling vs. usual care: US$115,397  Enhanced Brief Lifestyle Counselling vs. Brief Lifestyle Counselling: US$37,714 | 20% (Brief Lifestyle Counselling vs. usual care) and 47% (Enhanced Brief Lifestyle Counselling vs. usual care) | US$100,000 |
| Zhang 2021 | CUA | QALY | -- | -- | Using SF-6D instrument: US$96,458; Using Feeling Thermometer instrument: US$43,169; No difference in QoL when using HUI-2 or HUI-3 therefore undefined ICER. | NR | US$100,000 |
| **WMP - Other ^E^** | | | | | | | |
| Finkelstein 2014 ^F^ | CEA/CUA | QALY | -- | -- | Weight Watchers vs. control: US$34,630;  Qsymia vs. Weight Watchers: US$54,130;  Jenny Craig (average income) vs. Qsymia: US$588,620  Jenny Craig (high income) vs. Qsymia: US$377,760;  Orlistat: dominated;  Vtrim: extendedly dominated. | Weight Watchers dominates in 46% of simulations. Vtrim in 20% of simulations. | US$50,000 |
| Finkelstein 2019 ^F^ | CEA/CUA | QALY | -- | -- | Weight Watchers vs do nothing: US$30,071;  Qsymia vs Weight Watchers: US$117,219; Jenny Craig vs Qsymia: US$369,000;  Other interventions are dominated. | WW: 94% | US$50,000 |
| Krukowski 2011 | CEA | Life years gained | -- | -- | Internet-based: US$2160;  In-person: US$3306 | NR | NR |
| Tsai 2005 | CUA | QALY | -- | -- | US$1225 (dominant) | 78.6% 79.8% | US$100,000 US$150,000 |
| **WMP - Decision models** | | | | | | | |
| Avenell 2018  REBALANCE ^F^ | CUA | QALY | Lifetime | No | WMP1 vs baseline: £557; Surgery vs WMP1: £11,648; VLCD added to WMP1 was dominated; Look AHEAD and WMP2 were extendedly dominated. | NR | £20,000 |
| Gray 2018 | CUA | QALY | Lifetime | Yes | £2,150 | 100% | £20,000-30,000 |
| Kent 2019 | CUA | QALY | Lifetime | Yes | £12,955 | NR | £20,000 |
| Lewis 2014 ^F^ | CUA | QALY | 10 years | No | Results were presented for two groups.  BMI≥30 kg/m^2^:  Slimming World vs. no treatment: £5613;  Counterweight vs. Slimming World: £2618;  Weight Watchers vs. Counterweight: dominant;  LighterLife Total vs. Weight Watchers: £12,585.  BMI≥40 kg/m^2^:  LighterLife Total vs. no treatment: £4356;  GB vs. LighterLife Total: £20,505;  GBP vs. GB: £10,627 | NR | £20,000 |
| Meads 2014 | CUA | QALY | Lifetime | Yes | £6,906 | 68% | £20,000 |
| Miners 2012 | CUA | QALY | Lifetime | Yes | Only 2 ICERs reported for subgroup with BMI ≥35 kg/m^2^:  £151,142 (female without T2DM);  £232,911 (male with T2DM) | NR | £20,000 |
| Nuijten 2018 | CUA | QALY | 3 years | No | OPTIFAST vs no intervention: US$ 6,475 ^G^ | NR | US$50,000 |
| Radcliff 2020 | CUA | QALY | 5 years | No | Interventions (low, mod, high) versus control: dominant;  Mod versus low: US$107,895 | NR | NR |
| Thomas 2017 | CUA | QALY | 20 years | Yes | -£9358 ^H^ (less costly, more effective) | 93%  83% (cost-saving) | £20,000 |
| Trueman 2010 | CUA | QALY | Lifetime | No | -£473 (dominant) | NR | £20,000-£30,000 |
| Wilson 2015 | CUA | QALY | 5, 10 and 20 year time horizons (base-case not specified) | No | Results for those with morbid obesity ranged from: US$32,078 to US$335,952 | NR | US$50,000 |
| Wyke 2015 | CUA | QALY | Lifetime | Yes | £2,810 | 100% | £20,000-£30,000 |
| **Pharmacotherapy** | | | | | | | |
| Hertzman 2005 | CUA | QALY | 10 years | Yes | €13,125 | 90% | €22,000 |
| Lacey 2005 | CUA | QALY | 3 years | No | €16,900 | NR | NR |
| Veerman 2011 | CUA | DALY | Lifetime | No | AU$240,000 | NR | NR |

BMI, body mass index; CEA, cost-effectiveness analysis; CUA, cost utility analysis; DALY, disability adjusted life year; EE, economic evaluation; FFL, Fit For Life; GB, gastric banding; GBP, gastric bypass; HUI, Health Utilities Index; Mod, moderate; NR, not reported; Power+F, Positive Online Weight Reduction – face-to-face support; Power+R, Positive Online Weight Reduction – remote support; QALY, quality-adjusted life year; QoL, quality of life; SF-6D, Short-Form Six-Dimension; T2DM, type 2 diabetes mellitus; VLCD, very low calories diet; WMP, weight management programme.

^A^ The cost per QALY will be presented if available within the study.

^B^ The authors did however compare their cost per unit of effect with other published studies.

^C^ Probabilities of cost-effectiveness were read off the CEACs by the authors (see Figure 3 in the article).

^D^ For Power+F vs. control, the ICER (95% CI), incremental costs (95% CI) and QALYs (95% CI) were £1203 (–£35,636 to £38,403), £23 (95% CI: –£105 to £152) and -0.007 (95% CI: –0.030 to 0.014) respectively. For Power+R vs. control, the ICER (95% CI), incremental costs (95% CI) and QALYs (95% CI) were (–£26,621 to £27,765), -£36 (–£154 to £81) and -0.012 (–0.032 to 0.008) respectively.

^E^ Other study design is used to describe studies that are classified as neither RCT-based economic evaluations nor decision analysis models.

^F^ Fully incremental analysis on multiple treatments.

^G^ No ICER provided for the VLCD v surgery comparison.

^H^ Calculated by authors by dividing -£627 by 0.067 QALY gains.
